# Protein post-translational modification in SARS-CoV-2 and host interaction

**DOI:** 10.3389/fimmu.2022.1068449

**Published:** 2023-01-13

**Authors:** Nana Cheng, Mingzhu Liu, Wanting Li, BingYue Sun, Dandan Liu, Guoqing Wang, Jingwei Shi, Lisha Li

**Affiliations:** ^1^ China-Japan Union Hospital, Jilin University, Changchun, Jilin Province, China; ^2^ The Key Laboratory of Pathobiology, Ministry of Education, College of Basic Medical Sciences, Jilin University, Changchun, Jilin Province, China; ^3^ First Affiliated Hospital of Jilin University, Changchun, China; ^4^ Department of Pathogenobiology, The Key Laboratory of Zoonosis Research, Chinese Ministry of Education, College of Basic Medical Science, Jilin University, Changchun, China

**Keywords:** SARS-CoV-2, glycosylation, phosphorylation, acylation, ubiquitination, methylation, ADP-ribosylation

## Abstract

SARS-CoV-2 can cause lung diseases, such as pneumonia and acute respiratory distress syndrome, and multi-system dysfunction. Post-translational modifications (PTMs) related to SARS-CoV-2 are conservative and pathogenic, and the common PTMs are glycosylation, phosphorylation, and acylation. The glycosylation of SARS-CoV-2 mainly occurs on spike (S) protein, which mediates the entry of the virus into cells through interaction with angiotensin-converting enzyme 2. SARS-CoV-2 utilizes glycans to cover its epitopes and evade the immune response through glycosylation of S protein. Phosphorylation of SARS-CoV-2 nucleocapsid (N) protein improves its selective binding to viral RNA and promotes viral replication and transcription, thereby increasing the load of the virus in the host. Succinylated N and membrane(M) proteins of SARS-CoV-2 synergistically affect virus particle assembly. N protein regulates its affinity for other proteins and the viral genome through acetylation. The acetylated envelope (E) protein of SARS-CoV-2 interacts with bromodomain-containing protein 2/4 to influence the host immune response. Both palmitoylation and myristoylation sites on S protein can affect the virus infectivity. Papain-like protease is a domain of NSP3 that dysregulates host inflammation by deubiquitination and impinges host IFN-I antiviral immune responses by deISGylation. Ubiquitination of ORF7a inhibits host IFN-α signaling by blocking STAT2 phosphorylation. The methylation of N protein can inhibit the formation of host stress granules and promote the binding of N protein to viral RNA, thereby promoting the production of virus particles. NSP3 macrodomain can reverse the ADP-ribosylation of host proteins, and inhibit the cascade immune response with IFN as the core, thereby promoting the intracellular replication of SARS-CoV-2. On the whole, PTMs have fundamental roles in virus entry, replication, particle assembly, and host immune response. Mutations in various SARS-CoV-2 variants, which lead to changes in PTMs at corresponding sites, cause different biological effects. In this paper, we mainly reviewed the effects of PTMs on SARS-CoV-2 and host cells, whose application is to inform the strategies for inhibiting viral infection and facilitating antiviral treatment and vaccine development for COVID-19.

## 1 Introduction

Coronavirus disease 2019 (COVID-19), caused by severe acute respiratory syndrome coronavirus 2 (SARS-CoV-2), has become a global public health emergency (1). SARS-CoV-2 mainly spreads *via* respiratory droplets and adheres to the mucosal surfaces of the nasal cavity, oral cavity, and respiratory tract. SARS-CoV-2 can cause multi-system dysfunction, sequelae, and relapse in some cases (2). SARS-CoV-2 is a positive-sense single-stranded RNA virus belonging to the coronavirus family’s β-coronavirus genus, characterized by rapid variation, genetic diversity, and high prevalence (2). With the prevalence of the virus, SARS-CoV-2 continues to evolve. Currently, there are five SARS-CoV-2 variants of concern, (B.1.1.17) Alpha, (B.1.351) Beta, (P.1) Gamma, (B.1.617.2) Delta, and (B. 1.1.529) Omicron (3).

Post-translational modifications (PTMs) refer to covalent modifications after protein synthesis, catalyzed by specific enzymes, specifically on one or more amino acid residues of proteins in the form of covalent bonds adding corresponding functional groups, which play an important role in regulating protein solubility, activity, stability, subcellular localization, and mediating protein interactions. At present, common PTMs are glycosylation, phosphorylation, acylation, methylation, ADP-ribosylation, ubiquitination, and ubiquitin-like modification. In the process of virus-host interactions, viruses enhance viral replication, assembly, release and inhibit interferon responses through PTMs to facilitate virus proliferation and immune evasion. Instead, the host fights off viral infection by activating the autoimmune response and degrading the viral protein through PTMs. Compared with the wild-type (WT), mutations in various variants of SARS-CoV-2 lead to changes in PTMs of viral proteins, which affect various aspects of the virus, such as virulence and transmissibility. Instead, the host fights off viral infection by activating the autoimmune response and degrading the viral protein through PTMs (4).

This paper summarizes the PTMs that affect the pathogenicity of SARS-CoV-2, laying a foundation for a better intensive study of SARS-CoV-2 and its specific mechanisms affecting host cells. Conservative PTMs in SARS-CoV-2 and host interactions could promote the research and development of antiviral treatment and vaccines for COVID-19.

## 2 Glycosylation

### 2.1 On viral life cycle

**Figure 1:**
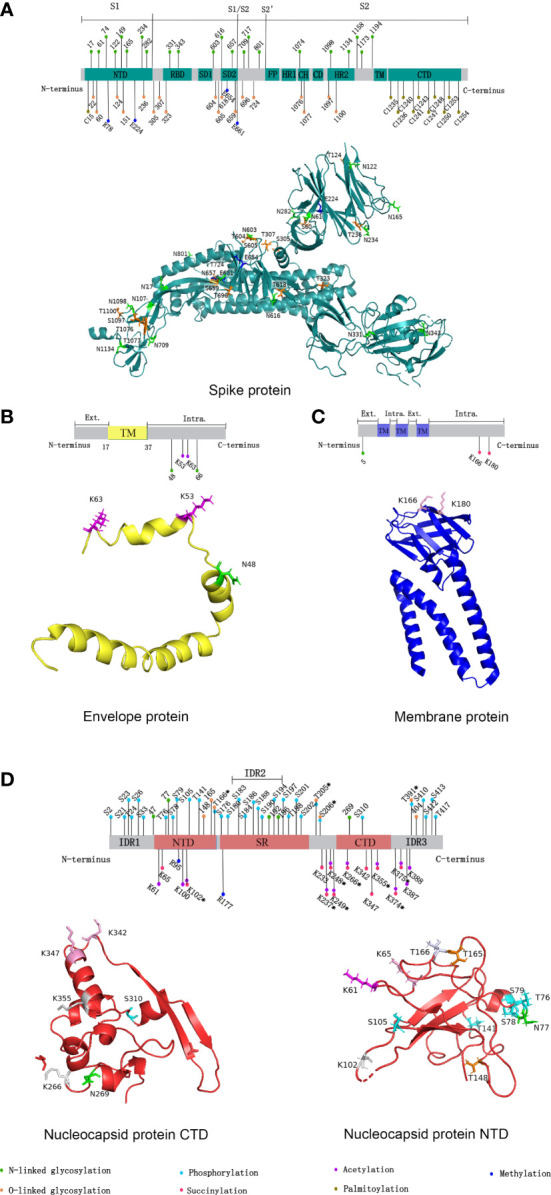
Distribution of SARS-CoV-2 structural proteins post-translational modification sites in 2D and 3D structural models. **(A)** Distribution of SARS-CoV-2 spike protein post-translational modification sites. **(B)** Distribution of SARS-CoV-2 envelope protein post-translational modification sites. **(C)** Distribution of SARS-CoV-2 membrane protein post-translational modification sites. **(D)** Distribution of SARS-CoV-2 nucleocapsid protein post-translational modification sites. In 2D structural models, the post-translational modifications represented by the different colors are shown in the figure legends. NTD, N-terminal domain; CTD, C-terminal domain; RBD, receptor binding domain; SD1/2, subdomain 1/2; FP, fusion peptide; HR1/2, heptad repeat region 1/2; CH, central helix; CD, connector domain; TM, transmembrane region; Ext., external; Intra., intravirion. (Sites with * indicate that various protein post-translational modifications have occurred.) In 3D structural models, different colors were used to represent different post-translational modifications occurring at a certain site. Green: N-linked glycosylation; Orange: O-linked glycosylation; Cyan: phosphorylation; Magenta: succinylation; Pink: acetylation; Blue: methylation; Gray 90: succinylation and acetylation; Bluewhite: O-linked glycosylation and phosphorylation. Due to the limitations of the 3D model, some sites could not be marked. We used the open source software PyMOL for mapping, and data were obtained from the RCSB PDB database (https://www.rcsb.org/).

Spike(S) protein mediates viral entry into host cells by binding to the cell surface receptor angiotensin-converting enzyme 2 (ACE2) (5). The S1 subunit binds to host cell receptors through the receptor binding domain (RBD), and the S2 subunit fuses with the host cell membrane (5). 17 O-linked glycosylation sites and 22 N-linked glycosylation sites can be detected in S protein, and the distribution of specific glycosylation sites on S protein is shown in **Figure 1A** (6). T323 and S325 in the RBD were associated with the process of binding to host receptors and membrane fusion respectively (6). Besides, N331 and N343 in the RBD can bind to the host lectin receptor, facilitating the entry of SARS-CoV-2 into the host cells (7). The glycosylation of S protein can mediate the folding of S protein, regulating the conformation of S protein, so that the membrane fusion can be initiated by host proteases to invade host cells (8). What’s more, SARS-CoV-2 blocks the innate immunity to the virus by utilizing host glycans to modify its surface proteins by glycosylation (9). Glycans form shields on the surface of the virus similar to that of endogenous host protein glycosylation, and sterically mask the polypeptide epitopes of the virus, then affect the recognition with receptors (9). The above process causes the viruses less recognized by the host immune system, evade the immune response, and strengthen the pathogenicity (9). Since the above mechanism and the glycan-dependent epitopes can cause a specific neutralizing antibody response, S protein is often used as an immunogen for vaccines, stimulating the body to produce neutralizing antibodies to prevent COVID-19 (8).

In SARS-CoV-2 variants, the mutation of S protein leads to increased virus transmissibility, immune evasion, and the resistance to neutralizing antibodies and vaccines. It’s worth noting that the N-linked glycosylation sites and O-linked glycosylation sites on S protein are highly conserved in the variants, except for the loss of the N17 site in the Delta variant and addition of the N20 site in the Gamma variant (3). However, although these sites are conserved, the glycosylation profiles between the variants are not same, in other words, the glycan patterns at S protein glycosylation sites have changed (3). The oligomannose-type glycans increase, while complex-type glycans decrease at glycosylation sites from Alpha variant to Delta variant (3). Compared with Delta, the oligomannose-type glycans of Omicron did not increase, but they were still more than the WT and other variants (3). The N501Y mutation in the RBD of S protein facilitates the interaction of the virus with ACE2 in Alpha variant, increasing viral infectivity and transmissibility (10). In addition, the D614G mutation occurred in Alpha variant (11). The D614G mutation is the most frequent, and it refers to the substitution of aspartic acid with glycine at position 614 of S protein (12). Compared with the WT, the D614G mutation decreases the amount of complex-type glycans by 45%, but increase the amount of oligomannose-type glycans by 33% (11). At the same time, the D614G mutation may influence the glycosylation near the N616 site, thereby influencing the conformational dynamics near the hydrophobic fusion peptide of S protein (13). Some studies showed that the D614G mutation changed the conformation of S protein, and enhanced its binding affinity with ACE2 (14). But most studies suggested that the D614G mutation made the shedding of S1 increase, which promoted membrane fusion (12). Compared with Alpha variant, the RBD of Beta variant also has two mutations, K417N and E484K, which make it completely different from the glycosylation pattern of Alpha variant (15). These differences enhance the binding affinity between the virus and ACE2 and immune evasion (15). Studies have found that mutations such as D614G, N501Y, T478K, and L452R have occurred in Delta variant, which eventually leads to an increase in the binding affinity of S protein to ACE2 (16). According to current research, a total of 37 mutations have occurred in Omicron variant, and its S protein retains all the above crucial mutations, making the virus more infectious (17). The functional examination of glycans differentially expressed on S protein of Omicron variant revealed that mutations in S protein increased the expression of complex-type glycans and promoted the binding of S protein to ACE2 (18). In addition, the higher abundance of sialic acid and galactose-containing glycan on S protein of Omicron variant significantly reduces the sensitivity of Omicron against S protein-neutralizing antibodies (18). Bioinformatics analysis showed that the P681R mutation of Delta can reduce the propensity for O-linked glycosylation near the S1/S2 cleavage site T678, promote the interaction between the virus and furin, and increase viral-cell fusion (19). In contrast, the N679K mutation at residue S683 in Omicron variant increases the propensity for O-linked glycosylation near the S1/S2 cleavage site, preventing recognition by proteases, thereby reducing virus entry into host cells and syncytia formation (19). However, the above conclusions still need to be confirmed by experiments. A recent mass spectrometry study showed that the RBD of S protein had a novel O-linked glycosylation site T376 in Omicron variant, compared with WT and Delta variant, but the effect of this mutation needs to be further researched (20).

Glycosylation is also present on proteins besides S protein in SARS-CoV-2. An N-linked glycosylation site N5 on M protein has been predicted, showed in **Figure 1C** (21). Highly glycosylated M protein maybe act as sugar transporters of eukaryotes (21). And M protein can assist S protein in membrane fusion and entry into host cells through glycosylation (22). Two glycosylation sites, N48 and N66, were also predicted on E protein, shown in **Figure 1B** (22). E protein can form glycans and bind to the host Toll-like receptor 2, which is necessary for viruses to induce cytokine storms in the body (22). Therefore, the binding of the E protein and Toll-like receptor 2 can promote the release of a variety of inflammatory cytokines in patients (22). Currently, five potential glycosylation sites on N protein have been predicted, including N47, N77, N192, N196, and N269, yet only N47 and N269 have been experimentally confirmed (23). In addition, MS showed a large number of O-linked glycosylation sites on N protein, including T148, T165, T166, T205, S206, T391, and S404 (23). The distribution of specific glycosylation sites is shown in **Figure 1D**.

### 2.2 On host

**Figure 2:**
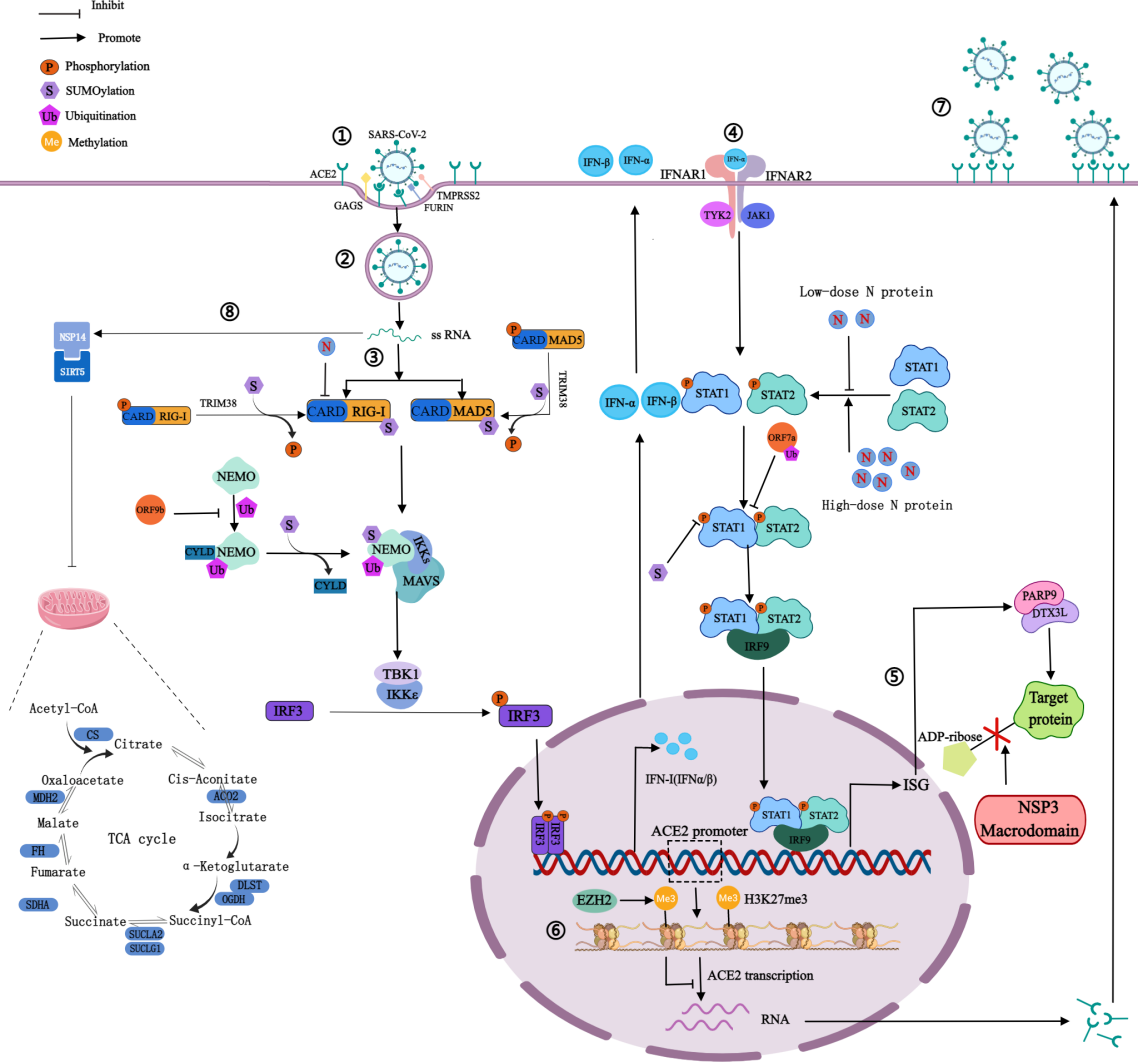
Mechanism of SARS-CoV-2 on PTMs of host proteins. ①Spike proteins recognize and bind to host cell surface receptors (ACE2, GAGS), TMPRSS2 and furin cleave the S1 and S2 subunits. ②Membrane fusion. ③The production of IFN: RIG-I and MAD5 recognize viral RNA and expose the CARD domain, through which RIG-I or MDA5 could interact with MAVS to activate the inhibitor of IκBϵ and TBK1, thereby phosphorylating IRF3 and phosphorylated IRF3 enters into the nucleus to activate IFN-I gene expression. ④The signaling process of IFN: Secreted IFN-α/β binds and activates JAK1 and TYK2, then they phosphorylate STAT1 and STAT2. Phosphorylated STAT1 and STAT2 can form heterodimers, which then bind to IRF9 to form a transcriptional complex IRF3 that translocates to the nucleus to induce the expression of ISG. ⑤NSP3 macrodomain reverses PARP9/DTX3L-dependent ADP-ribosylation of host proteins induced by IFN signaling. ⑥H3K27 can be methylated by EZH2 to form H3K27me3, then inhibits ACE2 expression in host cells. ⑦Highly expressed ACE2 receptors increase the risk of SARS-CoV-2 infection. ⑧NSP14 interacts with SIRT5 to reduce the level of protein succinylation in the host TCA cycle. The blue bars represent the metabolic enzymes in the TCA cycle.

ACE2, a type I transmembrane protein expressed by host epithelial cells, can regulate the renin-angiotensin system, and is mainly distributed in the heart and kidneys (9). ACE2 is a functional receptor for SARS-CoV-2 (9). SARS-CoV-2 recognizes and binds to ACE2 through the RBD of the S1 subunit, meanwhile, the glycosaminoglycans on the host cell membrane can promote the binding, and transmembrane serine protease 2 and fruin on the host cell membrane perform enzymatic cleavage at the connection region of S1 subunit and S2 subunit (5, 24). Then the S1 subunit sheds off, and the S2 subunit fuses with the host cell membrane, thereby invading host cells (**Figure 2**) (5, 24).

In patients who suffered from COVID-19, ACE2 is extensively glycosylated, and it 7 N-linked glycosylation sites and 1 novel O-linked glycosylation site can be detected on ACE2 (25). The extensive glycosylation of ACE2 has an impact on the attachment of SARS-CoV-2 to host cells (25). In glycans, sialic acid chains block the binding of ACE2 to RBD, especially N90, N322, and N546 (9, 26, 27). The glycosylation at N90 can interfere with the binding of the RBD to ACE2, acting as a steric hindrance and protecting host cells from viral infection (26). Conversely, glycans of N322 can bind tightly to the RBD, enhancing the formation of ACE2-RBD complexes (27). In general, the extensive glycosylation of ACE2 can regulate its binding to S protein, which in turn regulates the process of virus entry into host cells. But its regulatory effect is different according to the different glycosylation sites.

Similar to the glycosylation sites of S protein, the glycosylation sites of ACE2 are related to pathogenicity, infectivity, and immune evasion, and these glycosylation sites are important targets for the development of vaccines and antiviral therapy currently (9).

## 3 Phosphorylation

### 3.1 On viral life cycle

Phosphorylation can affect the activity of virus protein by inhibiting host translation and interfering with the formation of stress granules, which play an important role in translation control and antiviral immune response. In cells infected by SARS-CoV-2, high phosphorylation of N protein can be detected (28). There are 24 phosphosites on serine and 8 phosphosites on threonine of N protein, shown in **Figure 1D** (29, 30). The viscosity of N protein to RNA condensate decreases due to the abundance of phosphorylation in the serine-rich (SR) region, and low-viscosity phosphorylated condensate promotes viral replication and immune evasion (31). Unmodified N protein forms gel-like coagulants containing discrete RNP particles, which have high affinity mediated by multivalent RNA-protein, RNA-RNA, and protein-protein interactions, which is conducive to the compression of RNA genomes in virions (28). Phosphorylated N protein in the SR region blocks protein-protein interactions and forms dynamic liquid-like condensates, thus promoting the regulation of viral gene transcription (28). As the virus adapted, new variants of SARS-CoV-2 such as Alpha variant appeared, which significantly increased the subgenomic RNA and protein levels of all known innate immune antagonists of N protein, ORF9b, ORF6 (32). ORF9b plays an important role in the pathogenicity of Alpha variant (32). S53 on ORF9b was identified as an infection-driven phosphorylation site where serine formed a hydrogen bond with glutamic acid at E477 on TOM70, revealing the regulatory mechanism of ORF9b-TOM70 interaction: the binding of the phosphorylated ORF9b of variant to TOM70 was significantly reduced, and the binding of heat shock protein 90 (Hsp90) to TOM70 was not competitively inhibited (33). This suggests that the formation of the ORF9b-TOM70 complex is regulated by phosphorylation, while the binding of ORF9b depends on the state of its serine in S53 (33). The expression of ORF9b in human cells inhibits a signaling cascade involving the retinoic acid‐inducible gene-I (RIG-I), the mitochondrial antiviral signaling protein (MAVS), TOM70, TANK-binding kinase 1 (TBK1), and interferon regulatory factor 3 (IRF3), which eventually leads to a decreased expression of IFN-I response to RNA viral infection (34). After viral RNA recognition, RIG-I binds to MAVS, which in turn interacts with TOM70, causing Hsp90-bound TBK1 to associate with IRF3. ORF9b inhibits the combination of Hsp90 and TOM70, which may be a possible cause for decreased expression of IFN-I (34). Inhibiting phosphorylation of N protein could reduce intracellular viral RNA accumulation, viral load, and cell injury. Therefore, targeting GSK-3 may provide a way to prevent COVID-19 and future coronavirus outbreaks.

Three significant mutations were identified at amino acid residues 203-205 in the SR domain, including R203K+G204R (RG to KR mt) present in Alpha, Gamma, and Omicron variants; T205I present in Beta variant and R203M present in the Kappa and Delta variants (35). KR mt increases the phosphorylation of N protein accompanied by increased resistance to GSK-3 kinase inhibitors, resulting in enhanced viral replication. KR mt is more transmissible and adaptive than WT SARS-CoV-2 (36). However, KR mt does not alter the phosphorylation of mature virions (35). An R203A+G204A double alanine substitution mutant (AA mt) also shows increased replication, increased fitness, altered nucleocapsid phosphorylation, and similar resistance to kinase inhibition (35). KR mt may affect the interaction between N protein and the host 14-3-3 protein, which binds the SR domain in a phosphorylation-dependent manner, an interaction that is required for the cytoplasmic localization of the SARS-CoV-2 nucleocapsid (37). A 14-3-3 binding site contains the 203-204 motif of the SR domain, suggesting that KR mt may enhance infection by altering this interaction (37). In summary, KR mt and similar mutations enhance SARS-CoV-2 infection by increasing the phosphorylation of N protein by disrupting the ancestral “RG” motif.

### 3.2 On host

IFN-I is the first line of defense against viral infection, plays an important role in innate immunity, and has been used to treat patients infected with a variety of viruses. Low-dose N protein inhibits IFN-I response in the early stage of infection, resulting in immunosuppression (38). And in the later stage, high-dose N protein promotes the expression of IFN-I and inflammatory cells, resulting in an overactive immune response and cytokine storms (38). IFN-I has antiviral effects. In the first stage of viral infection, SARS-CoV-2 is recognized by host pattern recognition receptors, such as Toll-like receptor, NOD-LRR-containing receptor, RIG-I-like receptor, and C-type lectin receptor (38). The two major members of the RIG-I-like receptor family are RIG-I and melanoma differentiation-associated protein 5 (MDA5). Recognition of viral RNA exposes the amino-terminal caspase recruitment domain (CARD), through which RIG-I or MDA5 could interact with MAVS to activate the inhibitor of IκB kinase ϵ and TBK1, thereby phosphorylating IRF3, and phosphorylated IRF3 enters into the nucleus to activate IFN-I gene expression (39).In the second stage, secreted IFN-α/β binds and activates Janus kinase 1 and tyrosine kinase 2, then they phosphorylate the signal transducer and activator of transcription 1/2 (STAT1/2) of transcriptional proteins (39). Phosphorylated STAT1 and STAT2 can form heterodimers, which then bind to IRF9 to form a transcriptional complex. IFN-stimulated gene factor 3 translocates to the nucleus to induce the expression of the interferon-stimulated gene (ISG) and ultimately triggers an effective antiviral response (**Figure 2**). N protein dually regulates the phosphorylation and nuclear translocation of IRF3, STAT1, and STAT2 (38). Non-structural protein 1-16 (NSP1-16) can help coronaviruses antagonize the production of IFN-I. For instance, NSP6 interacting with TBK1 does not affect the phosphorylation of TBK1, but can inhibit the phosphorylation of IRF3; and NSP13 binds and blocks the phosphorylation of TBK1, inhibits the phosphorylation of IRF3 (39). The above pathways can lead to the reduction of IFN-β production, the inhibition of IFN-I signal transduction, the promotion of virus replication, acute respiratory distress syndrome, and extensive coagulation dysfunction in severe cases (39). SARS-CoV-2 was shown to be sensitive to IFN-I inhibition, suggesting that IFN-I may be used to treat COVID-19, especially in the early stage of infection (39).

## 4 Acylation

### 4.1 On viral life cycle

The main acylation modifications are succinylation, acetylation, and fatty acylation. SARS-CoV-2 protein succinylation occurs mainly on M protein and N protein, shown in **Figure 1C** and **Figure 1D**. With the assistance of M protein, N protein mediates the assembly of viral genomic RNA and nucleocapsid, which in turn affects viral replication (40). In M protein, there are 2 succinylation sites, both of which are located in the cytoplasmic domain. In N protein, there are 12 succinylation sites, 2 of which are located in the RNA binding domain (K65 and K102), and the remaining 10 are located in and near N protein dimerization domain, which may affect the formation of N protein dimer. Protein succinylation in SARS-CoV-2 is highly conserved, except for the K65 on N protein (40). Because its modification sites are highly conserved and have a very low rate of variability, while the nucleocapsid region of viral RNA has high density and frequency variation, broad-spectrum antiviral drug research targeting N protein succinylation has more wide application value (41).

Acetylation that occurs on N protein can affect the life cycle of the virus by regulating the affinity of N protein with the progeny virus genome and other proteins (42). In *in vitro* experiments, a total of 12 acetyl-lysine residues were identified in the N protein, showed in **Figure 1D**) (42). Among them, K61, K100, and K102 are located in the structural region of the interaction between N protein and the viral genome. The study speculated that it might be due to the acetylation of these three sites, especially the acetylation that occurs on K61, destroying the positive charge on N protein in the region that interacts with the viral genome, causing a decrease in its binding affinity with the viral RNA, then affecting the assembly of viruses (42). In addition, acetylation of K237, K248, and K249 may regulate the affinity of N protein to M protein (42). The acetylation of K53 and K63 at the C-terminus of E protein promotes the recognition and binding of the virus to bromodomain 1 (BD1) and bromodomain 2 (BD2) of transcriptional regulator bromodomain-containing protein 4 (BRD4) in host cells. The acetylated K63 can bind to BD1 and BD2, while the acetylated K53 can only bind to BD1 (43).

Palmitoylation is catalyzed by Asp-His-His-Cys (DHHC) palmitoyl acyltransferases, and DHHC is characterized by the zinc finger structure formed by the highly conserved DHHC motif as the catalytic center, so DHHC palmitoyl acyltransferases are also called zDHHCs. As the specific catalytic enzymes, the zDHHCs family can increase the palmitoylation level of S protein (44–46). Among them, zDHHC5, 9, and 20 may play a major catalytic enzyme role. For example, zDHHC9 can interact with S protein in the endoplasmic reticulum and Golgi to cause the palmitoylation of S protein, and the knockdown of the zDHHC9 gene can reduce the fusion and infection of SARS-CoV-2 (45). Compared with other β-coronavirus, the cysteine residues present most frequently on S protein of SARS-CoV-2, including 10 cysteine residues at its C-terminal (C1235, C1236, C1240, C1241, C1243, C1247, C1248, C1250, C1253, and C1254) and 1 cysteine residue at its N-terminal (C15), which are potential palmitoylation sites, showed in **Figure 1A** C-terminal and 1 cysteine residue at its N-terminal, which are potential palmitoylation sites (**Figure 1**) (44). Palmitoylation is important for S protein-regulated viral particle formation, viral entry into host cells, and viral infectivity (44, 45). High-level palmitoylation was identified on C1235, C1236, C1240, C1241, C1247, and C1248 (46). Among them, the palmitoylation of C1235, C1236, C1240, and C1241 has the most significant effect on the infectivity of viral particles by affecting the formation of S1/S2 subunits. In addition, their study suggested that C1253 and C1254 do not undergo palmitoylation (46).

Two myristoylation sites, 79 to 84,126 to 131, were found in the WT SARS-CoV-2 M protein. D3G mutation in the N-terminus of Omicron BA.1 M protein and D3N mutation in the N-terminus of Omicron BA.5 M protein may lead to myristoylation sites at positions 3-8. In the M protein V10A variant, a myristoylation site is present, 10. The D614G mutation was found in 84% of 6476 different S proteins, providing a myristoylation site at 614-619. Due to the absence of the acetyl group, the mutation prevents D614 from forming hydrogen bonds with T859 on chain A of S protein, and from forming salt bridges with T854 of the fusion peptide proximal region on chain B. The interaction of D614 with T859 and T854 enhanced the role of the fusion peptide proximal region in membrane fusion, which may affect the infectiveness of the virus (47, 48).

### 4.2 On host

Host protein succinylation is involved in many core energy metabolism pathways, including the tricarboxylic acid cycle (TCA), carbohydrate metabolism, fatty acid oxidation, etc. The virus can inhibit the process of nuclear export and mRNA translation in host cells so that the abundance of host intracellular proteins is greatly reduced, which leads to severe inhibition of the TCA cycle. NSP14 regulates host cell succinylation through interaction with sirtuin 5, which has a significant impact on the host cell TCA cycle (40). On the one hand, the levels of succinylation of rate-limiting enzymes associated with the TCA cycle increased, resulting in the inhibition of their activity (**Figure 2**) (40). On the other hand, the carbon sources of the TCA cycle are mainly glucose and glutamine, and in host cells infected with SARS-CoV-2, glutamine utilization is reduced (49). There are two main mechanisms: the abundance of glutaminase decreases, and glutaminase K164 succinylation leads to its own and glutaminase K158 ubiquitination, thereby reducing glutamine utilization (49).

BRD4 regulates cytokine storms and heart damage caused by COVID-19, and the binding of acetylated E protein to BRD2/4 can disrupt BRD-acetylated histone interactions and regulate viral beneficial protein expression in host cells (50). Inhibitors of BRD4, JQ1 or OTX015, can reduce the infectivity of SARS-CoV-2 in lung bronchial epithelial cells, suggesting that BRD4 and acetylation of E protein are potential targets for developing novel treatment strategies (43).

Myristoylated proteins may activate the major inflammatory factor nuclear factor-kappa B (NF-κB) and activate downstream inflammatory responses, which in turn leads to a series of immune responses (47).

As an important PTM, acylation is a key regulatory mechanism and a potential antiviral drug target, which can be used for the development of effective and safe antiviral drugs.

## 5 Ubiquitination and ubiquitin-like modification

### 5.1 On viral life cycle

SARS-CoV-2 uses the ubiquitin‐proteasome system to enhance the ability of ORF7a against IFN-I (51). ORF7a mainly binds to K63-linked polyubiquitin chains, achieves ubiquitination on K119, and blocks the phosphorylation of STAT2 to inhibit IFN-α signaling (52) (**Figure 2**). Ubiquitination of S protein may affect the structure of the S1 subunit and promote the binding of RBD to ACE2. Ubiquitination of N protein may promote the binding of the N-terminal domain to RNA and assist the viral RNA genome to assemble into a ribonucleoprotein complex. The complex is packaged into virions and promotes virus replication *in vivo* through interaction with the M protein (53).

### 5.2 On host

ORF9b targets NF-κB essential modulator (NEMO), a scaffolding component of the IκB kinase complex required for NF-κB activation, and interrupts K63-linked polyubiquitination of NEMO, thereby inhibiting IκB kinase α/β/γ -NF-κB signaling and IFN production (51) (**Figure 2**).

Ubiquitination is essential for activation and signal transduction during IFN-I production. The functional activity of RIG-I is closely related to K63 ubiquitination (53). Tripartite motif 25 (TRIM25) of E3 ubiquitin ligase enzyme interacts with RIG-I CARD (53). It catalyzes the K63-linked poly-ubiquitination of the K172 of RIG-I, which significantly enhances RIG-I activation (53). The E3 ubiquitin ligase enzyme TRIM31, which is recruited to the mitochondria after viral infection to interact with MAVS, catalyzes the K63-linked polyubiquitination of MAVS at K10, K311, and K461 sites, promoting the oligomerization of MAVS (53). However, K48 ubiquitination of RIG-I and MAVS leads to degradation, inhibiting IFN-I expression (53).

Ubiquitin-like proteins are similar in structure and function to Ub, including small ubiquitin-related modifier (SUMO), Neural Precursor Cell Expressed Developmentally Downregulated Protein (NEDD), and ISG15. SUMOylation of MDA5 and RIG-I catalyzed by the SUMO E3 ligase enzyme, TRIM38, is required for their dephosphorylation and activation upon viral infection (54). Conjugation of SUMO2/3 to MAVS induced by NSP5 induces subsequent activation of the NF-κB pathway, enhancing cytokine expression (54). The covalent binding of SUMO to STAT1 has been shown to reduce IFN-induced STAT1 phosphorylation and the production of IFN-γ (54). However, SUMO does not alter IFNα signaling, possibly because STAT2 is not affected by SUMO, which can compensate for the decrease of STAT1 phosphorylation (54). MDA5 CARD is ISGylated on K23 and K43, which promotes MDA5 activation by organizing MDA5 oligomers (55). The binding of ISG15 to MDA5 after viral infection is the trigger for promoting MDA5 oligomerization and antiviral immunity (55). It suggests that MDA5 ISGylation plays a role similar to the K63-linked ubiquitination of RIG-I.

SARS-CoV-2 encodes two proteases: papain-like protease (PLpro) and 3-chymotrypsin-like protease, which are a domain of NSP3 and NSP5, respectively (56). PLpro, a cysteine protease containing an N-terminal ubiquitin-like domain and a C-terminal ubiquitin-specific protease domain, has played two important roles in viral pathogenic mechanisms (56). First, PLpro recognizes the tetrapeptide LXGG motif between the viral proteins NSP1/2, NSP2/3, and NSP3/4 (57). It cleaves the N-terminal polyproteins (PP1a and PP1ab) to release NSP1, NSP2, and NSP3 and processes the remaining 13 nonstructural proteins with 3-chymotrypsin-like protease (57). Second, PLpro has the functions of deubiquitination and deISGylation, acting on host proteins to cleave Ub and ubiquitin-like proteins conjugates as well as ISG15 (57). PLpro dysregulates host inflammatory responses by deubiquitination and impairs host IFN-I antiviral immune response by removing ISG15 modification (56).

## 6 Methylation

### 6.1 On viral life cycle

The R95 and R177 on N protein of SARS-CoV-2 are methylated by the protein arginine methyltransferase 1 (PRMT1) under *in vitro* conditions, and the specific sites are shown in **Figure 1D** (58). Arginine methylation of N protein is associated with various pathological processes of the virus and host. It can inhibit the formation of stress granules, which in turn regulate virus replication and host cellular immune response (58). Ras-GTPase-activating protein SH3 domain binding protein 1 is a kind of stress granule, and the methylation of R95 on N protein can inhibit its formation, subsequently affecting the antiviral response (58). Besides, arginine methylation at R95 and R177 of N protein can promote the binding of N protein to the 5’-UTR of RNA (58). As mentioned above, N protein can also occur phosphorylation (28). Low phosphorylation of N protein can promote its binding to viral RNA. This suggested that the level of N protein binding to the 5’-UTR of viral RNA may depend on the balance of methylation and phosphorylation at specific sites of N protein. In addition, the methylation of N protein could promote the production of SARS-CoV-2 (58). When N protein methylation was inhibited, the viral load was reduced (58). Based on the above effects of N protein methylation, PRMT1 inhibitors may be a new target for the treatment of COVID-19 (58).

In addition to N protein, SARS-CoV-2 was also found to be methylated at multiple sites on S protein, including R78, E224, E654, and E661, and the distribution of sites is shown in **Figure 1A** (59).

### 6.2 On host

Protein methylation in host cells mainly occurs on histones, which is a research hotspot in epigenetics. In common, H3K4 and H3K79 methylation can promote gene transcription, in contrast, H3K9 and H3K27 methylation can inhibit gene transcription. In patients infected by SARS-CoV-2, histone methylation can regulate the gene expression of ACE2 and a variety of inflammatory cytokines (such as IFN, TNF, and ISGs).

Histone-lysine N-methyltransferase enzyme 2 (EZH2) can catalyze the methylation of H3K27 in the promoter region of the ACE2 gene to form H3K27me3 (60). In host cells, when EZH2 is deleted, the expression of H3K27me3 is decreased and the expression of ACE2 is increased (60). It may indicate that after SARS-CoV-2 infects host cells, H3K27 can be methylated by EZH2 to inhibit ACE2 expression in host cells, in turn, regulate host cell recognition and immune response to viruses, contributing to viral immune evasion (**Figure 2**) (60). The more ACE2 is expressed, the greater the risk of SARS-CoV-2 infection (60). Besides, multiple genes which are positively associated with ACE2 expression were regulated by lysine demethylase 5B (KDM5B) through pathway enrichment analysis (61).H3K4me3 can be demethylated by KDM5B, then hypomethylated histones can regulate the transcription of the ACE2 gene (61). KDM5B was considered a potential regulator of ACE2 expression (61). Histone methylation can also regulate the expression of inflammatory genes, which is closely related to the innate immune response after viral infection (61). In peripheral blood mononuclear cells from COVID-19 patients, the increase of H3K27me3 in the promoter region of miR-146a inhibited miR-146a expression (62). MiR-146a directly targets IL-6 and IL-6 can promote monocyte proliferation (62). Therefore, the increase of H3K27me3 in the promoter region of miR-146a may inhibit the proliferation of monocytes and promote immune evasion.

Histone methylation can regulate the expression of genes corresponding to a variety of inflammatory cytokines in host cells, and histone lysine methyltransferases can regulate the function of immune cells. At present, histone lysine methyltransferase inhibitor has emerged as a new therapeutic target to restore the immune imbalance caused by a cytokine storm in patients (63).

In addition, ACE2 has been found to contain multiple highly methylated sites, especially E57, K68, and E329, which are all fully methylated near the binding sites of ACE2 and RBD (59).

## 7 ADP-ribosylation

At present, research on ADP-ribosylation of proteins after SARS-CoV-2 infection focuses on NSP3. The production of IFN can induce host proteins to occur PARP9/DTX3L-dependent ADP-ribosylation (64). However, the ectopic expression of the NSP3 macrodomain in SARS-CoV-2 can reverse this modification, and hydrolyze the final product of the IFN signaling process (64). The above process inhibits the cascade immune response with IFN as the core, rather than directly inhibiting the IFN reaction itself, so that viruses can replicate in host cells (**Figure 2**). The target of NSP3 may be ADP-ribosylation on PARP14-dependent STAT1, which in turn affected ISG transcription and IFN expression, causing severe cytokine storms, but this prediction has not been directly supported by evidence (65). NSP3 macrodomain is a potential drug target, and NSP3 macrodomain inhibitors may become a new treatment for COVID-19.

## 8 Conclusion and future outlook

PTMs play a pivotal role in regulating the replication, infectivity, and pathogenicity of SARS-CoV-2 in the process of virus infection and host response. A summary of the effects of PTMs of SARS-CoV-2 viral proteins on the virus and the host is provided in **Table 1** and **Table 2**. SARS-CoV-2 successfully enters host cells, evades the host immune response, helps the viral RNA to be efficiently translated and copied, increases the viral load in host cells, and affects the metabolism of host cells through PTMs. PTMs are induced at an early stage by cascading reactions when the virus enters host cells. After SARS-CoV-2 enters host cells, the viral proteins interfere with the host immune response by inhibiting PTMs of the host. On the other hand, viral proteins specifically bind to enzymes in host cells, trigger PTMs of the host cells, change the structure and property of the host proteins, inhibit the host protein activity, evade the host immune response, and increase the viral virulence.

**Table 1:** Effects of PTMs of SARS-CoV-2 proteins on the virus.

Protein modification		Related protein	Impacts on viruses
glycosylation		S protein	Promotes the binding of S protein and ACE2, masks antigenic epitopes through glycans, and evades the host immune response (9).
		E protein	Binds to the Toll-like receptor2 in the host through glycans, and promotes the release of inflammatory cytokines (22).
		M protein	May form sugar transporter proteins, help S protein enter into host cells, and mediate the assembly of viruses (66).
		N protein	/
		ORF3a	May mediate that viruses enter into and release from host cells (67).
phosphorylation		N protein	Enhances its affinity for RNA binding, promotes viral replication and viral particle assembly, as well as interferes with the localization of stress granules in host cells (28, 31).
		ORF9b	Interacts with TOM70 and inhibits the host signaling cascade that produces IFN-I (34).
acylation	succinylation	M protein	Affects the virus replication by influencing the assembly of viral genomic RNA and nucleocapsid (40).
		N protein	
	acetylation	E protein	Interacts with BRD2/4 and influences host immune responses (50).
		N protein	Regulates the affinity of N protein to progeny viral genomes and other proteins (42).
	palmitoylation	S protein	Affects the production of virus particles, entry into host cells, and infectivity (44, 45).
	myristoylation	S protein	Affects the infectivity of the virus (47).
		M protein	/
ubiquitination		NSP3	PLpro is a domain of NSP3 that dysregulates host inflammation by deubiquitination (56).
		ORF7a	Inhibits host IFN-α signaling by blocking STAT2 phosphorylation (52).
ubiquitin-like modification		NSP3	PLpro impinges host IFN-I antiviral immune responses by deISGylation (56).
methylation		S protein	/
		N protein	Inhibits the formation of host stress granules, promotes the binding of N protein to the 5’-UTR of RNA, and facilitates the production of SARS-CoV-2 (58).
ADP-ribosylation		NSP3	NSP3 macrodomain has ADP-ribosylhydrolase activity, reverses PARP9/DTX3L-dependent ADP-ribosylation of host proteins induced by IFN signaling, and inhibits cascade immune response with IFN as the core (64).

**Table 2:** Effects of post-translational modifications of SARS-CoV-2 proteins on the host.

Protein modification		Impacts on the host
**glycosylation**		The glycosylation sites of ACE2, such as N90 and N322, can regulate its binding to S protein, which in turn regulates the process of virus entry into host cells (25–27).
**phosphorylation**		Viral proteins, such as N protein and NSP6, can block the phosphorylation of IRF3 and TBK1 in the JAK-STAT signaling pathway and inhibit the host immune response (38, 68, 69).
**acylation**	succinylation	NSP14 interacts with sirtuin5 to regulate the succinylation of host protein and inhibit the TCA cycle of the host, thus affecting the energy supply system of the host (40).
	acetylation	The binding of acetylated E protein to BRD2/4 disrupts the interaction of BRD-acetylated histone and regulates the expression of beneficial viral proteins in host cells (50).
	palmitoylation	/
	myristoylation	Myristoylated proteins may induce a series of inflammatory reactions in host cells by activating NF-κB (47).
**ubiquitination**		ORF9b can block the ubiquitination of host NEMO, thereby inhibiting IFN production (51). PLpro, a domain of NSP3, can modulate the host inflammatory response by deubiquitination, deSUMOylation, and deISGylation, thereby impairing the host IFN-I antiviral response (56, 57).
**ubiquitin-like modification**		
**methylation**		Host histones can be methylated or demethylated by enzymes such as EZH2 and KDM5B to regulate the gene expression of ACE2 and various inflammatory cytokines, thereby regulating the host antiviral immune response (60, 61, 70).
**ADP-ribosylation**		Host proteins can be induced by IFN to occur PARP9/DTX3L-dependent ADP ribosylation, thereby causing a cascade immune response with IFN as the core and playing an antiviral role (64).

On S protein, the main PTMs are glycosylation, palmitoylation, myristoylation, and methylation. They not only lead to the conformational change of S protein and enhance the affinity with ACE2, but also promote the membrane fusion process. As a result, the above progress helps the virus enter the host cell and affects the production of virus particles, which enhances viral infectivity. At the same time, glycans formed by glycosylation can mask the epitopes of virus antigens and evade the host immune response. By interacting with host toll-like receptor 2 and BRD2/4, glycosylated and acetylated E protein induces the release of downstream inflammatory cytokines and influences the host immune response. Glycosylation, succinylation, and myristoylation occur on M protein. Modified M protein may form sugar transporters that help S protein enter host cells and affect viral replication by influencing viral genomic RNA and nucleocapsid assembly. A variety of PTMs occur on N protein, including phosphorylation, succinylation, acetylation, methylation, and glycosylation. The synergistic effect of various PTMs promotes the binding of N protein to RNA 5 ‘-UTR and improves its affinity with RNA, which mediates the assembly of viral genomic RNA and nucleocapsid. In addition, the modified N protein also interferes with the localization and formation of stress granules in host cells.

In various variants of SARS-CoV-2, the amino-terminal composition is altered due to the change of the virus gene, and then the PTMs on the protein are varied so that the viral transmissibility and virulence are shifted. For example, by changing the glycan pattern of S protein, such as the abundance of complex-type glycans and oligomannose-type glycans, the D614G mutation may alter the conformation of S protein, promoting the membrane fusion, thereby making the variants more infectious and transmissible. Currently, most vaccines are designed to target S protein. As a result, mutations occurring on the surface of S protein may affect the efficacy of the vaccine. Thus, it is important to examine the effects of different mutations on vaccine efficacy.

At present, further studies on the effects of PTMs on SARS-CoV-2 and host cells are needed. First, the specific PTM sites on viral proteins need to be further studied and validated *in vivo*. For example, the acetylation sites on N protein need to be further verified *in vivo*. Besides, the study of SARS-CoV-2 glycosylation has focused on S protein, and few studies have been done on whether there are glycosylation sites on other structural proteins. N protein has been considered to be completely non-glycosylated, but recently there have been studies using mass spectrometry to predict multiple glycosylation sites on N protein, such as N47, N77, etc. However, the above research conclusions need to be confirmed by further experiments. Second, the specific effects and mechanisms of PTMs at each site are still incomplete. For instance, the D614G mutation, common in various SARS-CoV-2 variants, is believed to affect the glycosylation of S protein N616 site, which changes S protein from a closed conformation to an open conformation, but whether the D614G mutation affects the S protein binding to ACE2 is controversial. Therefore, the molecular mechanism of how the D614G mutation affects the glycosylation of S protein and enhances virus transmissibility and immune evasion has been a current research hotspot. Third, the effect of viral infection on PTMs of host proteins and the effect of PTMs of host proteins on the pathophysiology of host cells need to be further explored. Fourth, the effect of mutations on vaccine efficacy is still unknown. According to theoretical speculation, the mutation of D614G is located inside the structure of S protein and will not cause changes in the epitope and surface structure of S protein, but the mutation occurring in the RBD region of S protein has a high probability of affecting the efficacy of the vaccine. It all depends on further tests.

In this paper, we reviewed the effects of several common PTMs in SARS-CoV-2 and the host, briefly described the mechanism of PTMs assisting in virus pathogenesis or immune evasion, and summarized some modification sites or pathways, which may become important targets for vaccine development and have great significance for the future treatment of COVID-19.

## Author contributions

All authors contributed to the article and approved the submitted version. LL, JS and GW designed and polished the paper.
